# Strategies to Improve the Safety of iPSC-Derived β Cells for β Cell Replacement in Diabetes

**DOI:** 10.3389/ti.2022.10575

**Published:** 2022-08-24

**Authors:** Silvia Pellegrini, Valentina Zamarian, Valeria Sordi

**Affiliations:** Diabetes Research Institute, IRCCS San Raffaele Hospital, Milan, Italy

**Keywords:** cell therapy, safety, type 1 diabetes mellitus, induced pluripotent stem cells, beta cells

## Abstract

Allogeneic islet transplantation allows for the re-establishment of glycemic control with the possibility of insulin independence, but is severely limited by the scarcity of organ donors. However, a new source of insulin-producing cells could enable the widespread use of cell therapy for diabetes treatment. Recent breakthroughs in stem cell biology, particularly pluripotent stem cell (PSC) techniques, have highlighted the therapeutic potential of stem cells in regenerative medicine. An understanding of the stages that regulate β cell development has led to the establishment of protocols for PSC differentiation into β cells, and PSC-derived β cells are appearing in the first pioneering clinical trials. However, the safety of the final product prior to implantation remains crucial. Although PSC differentiate into functional β cells *in vitro*, not all cells complete differentiation, and a fraction remain undifferentiated and at risk of teratoma formation upon transplantation. A single case of stem cell-derived tumors may set the field back years. Thus, this review discusses four approaches to increase the safety of PSC-derived β cells: reprogramming of somatic cells into induced PSC, selection of pure differentiated pancreatic cells, depletion of contaminant PSC in the final cell product, and control or destruction of tumorigenic cells with engineered suicide genes.

## Introduction

In patients with type 1 diabetes (T1D), glycemic control can be reestablished by allogeneic islet transplantation. However, this approach is severely limited by the scarcity of organ donors. A new source of insulin-producing cells would significantly increase the possibility of cell therapy becoming a broad and standard therapy for the treatment of all diabetic patients. Pluripotent stem cells (PSC), such as embryonic stem cells (ESC) and induced pluripotent stem cells (iPSC) derived from somatic cell reprogramming, can differentiate *in vitro* into insulin-producing cells with established protocols that recapitulate embryonic pancreas development. In the first clinical trials, PSC-derived β cells were transplanted into patients with type 1 diabetes (NCT03163511, NCT02239354, and NCT04786262). In this context, the safety of the final cellular product in developing PSC derivatives for transplantation prior to implantation is crucial (1,2). Indeed, not all PSC reach complete differentiation into functional β cells *in vitro*, and a fraction of the cells may remain undifferentiated, exposing recipients to the risk of teratoma formation post-transplantation.

The most common method for determining pluripotency is the teratoma formation model, which employs immunodeficient animal models, in which pluripotent cells develop into teratomas formed from all three germ layers. A direct comparison of the teratoma formation capacity between ESC and iPSC revealed that iPSC form teratomas more efficiently and quickly than ESC (3). It is also likely that the extended *in vitro* culture and manipulation of PSC facilitates accumulation of genetic lesions (4–6), as well as genetic and epigenetic abnormalities during reprogramming to pluripotency (1,7). Even a very small contaminant at the end of differentiation constitutes a risk. It has been found that as few as two ESC colonies implanted into immunodeficient mice can result in teratoma formation; when the clumps were trypsinized to single-cell suspensions before injection, 245 cells were sufficient to form teratomas after 10 weeks (8). Several groups have reported the formation of teratomatous tissue elements in grafts when not purified PSC-derived pancreatic endoderm cells were infused in mice (9–13). Although recent protocol refinements have reduced this risk and increased the percentage of mature β cells obtained, there remains a need to control contaminant pluripotency in β cells. Therefore, the therapeutic applications of PSC-differentiated derivatives require strategies for the control of innate tumorigenicity and the malignant transformation of inappropriately differentiated cells.

The foreseeable implementation of stem cell-based therapies for the treatment of thousands of patients requires extreme caution, as only a single case of stem cell-derived tumors can set the field back several years. The first published data on patients with T1D transplanted with PSC-derived pancreatic progenitors showed that the transplanted cells did not form tumors, but only a percentage of the implanted cells survived and secreted C-peptide (14,15). Therefore, whether a greater number of implanted and engrafted cells can give rise to teratomas remains unclear. In this review, approaches to increase the safety of PSC-derived β cells are discussed, which can be summarized in four different strategies:

(1) The generation of safe iPSC using advanced techniques for cell reprogramming that conjugate non-integrating delivery of Yamanaka’s factors and high efficiency.
(2) The selection of pure differentiated cells based on specific β cell or pancreatic precursor markers, allowing for the selection of target cells only.
(3) The depletion of contaminant PSC in the final cell product, using chemical inhibitors or the selective killing of contaminant stem cells.
(4) The control of tumorigenic cells with suicide genes, in which stem cells are harbored with one or more suicide gene cassettes, resulting in cell death in the presence of specific prodrugs.

Herein, these approaches are discussed with the belief that the best results will most likely be obtained using a strategy that combines the choice of the safest PSC source, the selection of the cellular product, and protection via the use of safety switches.

## Generation of the Safest Induced Pluripotent Stem Cell

IPSC can be derived from any individual, with the advantages of possessing the same plasticity as ESC while avoiding the ethical problems arising from the use of human embryos. For these reasons, iPSC are considered valuable tools in regenerative medicine, disease modelling, and drug discovery. IPSC are generated through the genetic reprogramming of adult somatic cells; however, inserting reprogramming factors into adult cells raises safety issues. In fact, iPSC reprogramming was originally obtained by the overexpression of four transcription factors (Oct4, Sox2, Klf4, and c-Myc), subsequently denoted as the “Yamanaka factors,” with a retroviral delivery system in murine and human fibroblasts (16,17). The disadvantage of this original reprogramming method from a translational perspective is that reprogramming vectors are randomly integrated into the genome of transduced cells, leading to risks including teratomas and genomic instability (18,19). Several integration-free alternative methods have been developed and tested to overcome these safety issues. Without the intention of describing all the reported reprogramming techniques and how these have changed since the discovery of iPSC 15 years ago, this review focuses on the optimal reprogramming for the safe application of iPSC in the field of cell replacement therapies.

The most important factor that should be considered for the reprogramming of donor cells includes the “footprint” that a particular method deposits in the reprogrammed cell type. Within cellular replacement therapy, iPSC should have no footprint and no residual transgene sequences of the reprogramming vectors in the final iPSC product. This can be achieved using methods of transfection with episomal plasmids or minicircles, infection with non-integrating Sendai Virus (SeV) or adenovirus, transfection with synthetic mRNA/miRNA, or transposition with the piggyBac transposon, all of which leave no traces of the integration of the transgenes in the genome of reprogrammed iPSC (18). Alternatives include the use of lentiviruses and retroviruses that, with an additional step after reprogramming, allow for the excision of the transgene, such that only a small portion of the reprogramming vector remains integrated in the iPSC genome. Combining this characteristic of the “zero” footprint with an acceptable level of efficiency and the need for commercially available easy-to-use reagents that meet good manufacturing practice (GMP) standards, episomal plasmids and Sendai virus are currently the best choices for generating iPSC for projects with translational endpoints (20,21).

At present, the most commonly used strategy for reprogramming with SeV involves the delivery of Oct4, Sox2, Klf4, and L-Myc genes (22). Sendai virus is an enveloped virus with a single-chain RNA genome, and its two main characteristics make SeV the most attractive system for reprogramming. First, it can infect a wide range of cell types, infecting cells by attaching itself to the sialic acid present on the surface of multiple somatic cells, including PBMC, CD34^+^ cells, and T cells. Second, SeV vectors are made of RNA and remain in the cytoplasm, ensuring that they do not integrate into the host genome or alter the genetic information of the host cell (22–24). Importantly, however, in the most recent version of SeV, the F gene, responsible for fusion protein expression, was deleted, and new temperature sensitivity mutations to the polymerase-related genes were added to counteract the formation of non-transmissible virus-like particles. These modifications prevent transmission and limit the propagation of reprogramming vectors, helping to clear the virus faster after reprogramming and reducing cytotoxicity to cells (25).

## Selection of Pure Differentiated Cells

IPSC reprogramming using a safe method represents a step towards guaranteeing a safer cellular product. However, it does not protect completely against the risk of tumorigenesis. Indeed, although multistep differentiation protocols lead to the *in vitro* production of functional insulin-producing cells from PSC (26–30), the differentiated cultures can also contain undesirable proliferating cell types, such as residual pluripotent cells, which can jeopardize graft safety. The most intuitive and reasonable approach for the selection of β cells, capable of purifying the cell preparation to be transplanted while eliminating unwanted unsafe cells, is the positive selection of the target cells. This approach is mainly mediated by antibodies that bind to specific proteins expressed on the surface of pancreatic cells. Two main strategies have been developed: the selection of pancreatic endoderm (PE) progenitors and the selection of mature β cells. In both cases, it has been necessary to rely on transcriptomic and proteomic studies aimed at describing specific markers (31–35). Despite efforts to characterize insulin-producing cells and their precursors, there are currently no universally shared surface markers of these cell types. Finding endodermal-specific markers is not an easy task and requires the careful analysis of differentiating cells during embryogenesis. One elegant study mined microarray gene expression data from early murine embryos to identify two PE-specific cell-surface proteins (31,32), namely PDGFRα and Lrp2. However, the presence of RNA during development does not always correlate with the presence of the protein (33). Another study revealed that of all protein classes examined, cell-surface proteins in particular showed a poor correlation between protein and RNA abundance when comparing cell types (34). Therefore, RNA expression may be an unreliable predictor of specific surface protein expression; thus, proteomic approaches are needed to identify protein markers that can distinguish cell types in developing embryos. In a pioneering study, Rugg-Gunn et al. developed a direct proteomic approach to explore the cell-surface proteome for developmental lineages using affinity labelling and mass spectrometry. They identified molecules with potential importance in the separation and migration of endoderm, which allowed for the prospective isolation and characterization of viable PE directly from mouse blastocysts (35). The results obtained in the mouse model highlighted a strategy with which to find specific lineage markers for transfer into human cells.

An early work aimed at identifying β cell markers useful for the purification of cells during the last stages of differentiation from stem cells was published in 2011 by the group of scientists of Viacyte Inc., who proposed three proteins as specific to different stages (9). Using a flow cytometry-based screening of commercial antibodies, the researchers identified cell surface markers for the separation of pancreatic cell types derived from human ESC. In particular, CD200 and CD318 were used as markers of endocrine cells. However, when these sorted cells were implanted *in vivo*, they gave rise mainly to glucagon-positive cells. In contrast, CD142, also known as a tissue factor, was found to enrich PE cells, which give rise to all pancreatic lineages, including functional insulin-producing cells after transplantation into mice. In fact, the transplantation of CD142 sorted cell aggregates gave rise to functional, glucose-responsive, insulin-secreting cells *in vivo*, whereas the transplantation of unenriched material resulted in teratomatous graft rates of 45% (9). The main limitation of the use of CD142 as a selection marker for pancreatic differentiation is its low specificity. Several other cell types, including endothelial cells, monocytes, macrophages, and platelets, express CD142.

In the same year, a study reported CD24 as a new surface marker for pancreatic progenitors differentiated from human ESC (36). CD24 is a sialoglycoprotein normally expressed on mature granulocytes and B cells that modulates growth and differentiation signals in these cells. In this study, CD24 was identified as a positive marker of pancreatic progenitors by co-staining for PDX1 and a panel of cell surface antigens at the pancreatic progenitor stage of human ESC differentiation. CD24^+^ cells co-expressed most of the key transcription factors of pancreatic progenitors, and the expression of important pancreatic genes was significantly enriched in CD24^+^ cells compared with CD24^−^ cells. Notably, CD24^+^ cells could differentiate into insulin-producing cells, but CD24^−^ negative cells could not. As in the case of CD200 and CD318, the use of CD24 did not include a follow-up to purify differentiated cells, and to date, CD24 plays a role mainly as a cancer stem cell marker for ductal adenocarcinoma (37).

A substantial new impetus to the surface marker-based selection approach came when three major papers on the GP2 protein were published in 2017. In the first study, the researchers performed microarray analysis to compare the gene expression pattern of PDX1^+^/NKX6.1^+^ pancreatic progenitors with that of PDX1^+^/NKX6.1^-^ cells and identified progenitor-specific cell surface markers (38). CD142 and CD200, two cell surface markers previously shown to enrich pancreatic endoderm cells and endocrine progenitors (9) were expressed in both cell populations. In addition, the researchers identified a cell surface maker, glycoprotein 2 (zymogen granule membrane GP2), which was enriched in the PDX1^+^/NKX6.1^+^ cell population obtained from PSC differentiation and fetal pancreas (38), which could potentially be used for the isolation of pancreatic progenitors. Furthermore, the researchers showed that the isolated GP2^+^ progenitors efficiently differentiated into glucose-responsive insulin-producing cells *in vitro*. Another study reported that GP2^+^ cells, obtained from the human pancreas at 7 weeks of development, purified and cultured *in vitro*, might give rise to acinar cells, in which GP2 is upregulated, as well as ductal and endocrine cells, in which GP2 is downregulated or silenced. In this study, human fetal pancreatic differentiation was reconstructed using GP2 in combination with CD142 to mark pancreatic progenitors, which could give rise to GP2^hi^CD142^+^ acinar cells or enter the endocrine pathway and express NEUROG3 by turning off GP2 and CD142 (39). At the same time, Cogger et al., in Canada, used a proteomics approach to phenotypically characterize pancreatic progenitors derived from PSC and distinguish these cells from other populations during differentiation (40). In addition, GP2 has been identified as a specific cell surface marker for pancreatic progenitors (40). In the developing human pancreas, GP2 is co-expressed with the endocrine key transcription factors NKX6.1 and PTF1A. In addition, isolated PSC-derived GP2^+^ cells were shown to generate β cells more efficiently than GP2^−^ and unsorted populations, decreasing the percentage of unwanted PSC-derivatives, consequently increasing the safety of the final cell product. This last point was taken up and confirmed by a very recent study by the same group, wherein they showed that sorted GP2-expressing pancreatic progenitors give rise to all endocrine and exocrine cells *in vivo*, including functional β cells, without influencing the endocrine-to-acinar ratio within the graft, and that GP2 sorting prevents teratoma formation *in vivo*. These findings support GP2 as a candidate marker for cell selection with potential for clinical use (41).

Another surface marker for differentiating pancreatic cells, but at an earlier stage of differentiation, was recently reported: the CD177/NB1 glycoprotein. This glycoprotein was identified as a novel surface marker to isolate pancreatic progenitors from definitive endoderm cells derived from human PSC. Isolated CD177^+^ definitive endoderm differentiated more homogeneously into pancreatic progenitors and into more functionally mature and glucose-responsive β cells than cells from unsorted differentiation cultures (42). Therefore, CD177 is a promising marker for cell selection during pancreatic differentiation to improve differentiation efficiency, but it is likely to be an early marker to purify progenitors for safety purposes. It is worth noting the work by Melton’s team, whose research resulted in a differentiation protocol to produce β cells that are now being transplanted into patients in an ongoing clinical trial (NCT04786262). In a study published in Nature in 2019, Veres et al. used a strategy for endocrine cell enrichment based on single-cell dissociation followed by controlled re-aggregation (43,44)*.* This technique was coupled to the selection of cells with a marker, CD49a/ITGA1, identified by single-cell transcriptomic analysis (45). Anti-CD49a staining and magnetic microbead labelling allowed for the efficient sorting of stem cell-derived β cells. This method produced clusters containing up to 80% β cells from embryonic and induced pluripotent stem cell lines. These highly purified β cells were responsive to glucose *in vitro* and had increased stimulation indices compared to unsorted, re-aggregated islets in both static and dynamic glucose-stimulated insulin secretion (GSIS) (45). It is reasonable to assume that this purification level reduces the risk of non-pancreatic contaminants in the final cellular product, thereby increasing its safety. In 2020, one study reported an antibody panel against cell surface antigens to enable the isolation of highly purified endocrine subsets from mouse islets, and CD71 was used as a specific marker of adult β cells. CD71 is a transferrin receptor that mediates the uptake of transferrin-bound iron whose expression is regulated in a glucose-dependent manner. β cells were also found to express high levels of several other genes implicated in iron metabolism, and iron deprivation significantly impaired β cell function (46). These findings have interesting implications on iron metabolism in β cell function, as well as for the discovery of CD71 as a novel surface marker of β cells, at least in mouse islets.

Another potential marker for the identification of adult β cells is CD81/TSPAN28. In a recent study, the researchers performed single-cell mRNA profiling of early postnatal mouse islets, re-analyzed several single-cell mRNA sequencing datasets from mouse and human islets, and complemented the findings by testing iPSC-derived endocrine cells, Min6 insulinoma, and human EndoC-βH1 β cell lines (47). They found that CD81 marks immature β cells in healthy islets and labels dedifferentiated β cells in metabolically stressed environments, such as during diabetes progression. Since it is possible that β cells derived from stem cells share some features of dedifferentiated or immature cells, CD81 could be a valuable tool for targeting β cells and purifying them from the bulk of progenitors and non-β cells present in the final cell product of differentiation. Since CD81 likely marks immature β cells, with reduced levels of expression associated with increased gene regulatory networks involved in maintaining β cell maturation, it could be used to select differentiating cells at the stage of immature β cells, when Nkx6.1 is upregulated, but cells do not yet secrete insulin (47).

Another possibility for the efficient purification of insulin-positive cells involves cell sorting based on the expression of insulin at the immature β-like stage. However, this purification method, successfully reported in some studies (30), requires the cell sorting of a genetically modified human ESC line in which a green fluorescent protein (GFP) reporter gene was inserted into the endogenous human insulin locus. One paper using a new approach was published this year, in which the researchers describe the generation of an array of monoclonal antibodies against cell surface markers that selectively label stem cell-derived islet cells (12). High-throughput screening identified promising candidates, including three clones that marked a high proportion of endocrine cells in differentiated cultures. These three antibodies, 4-2B2, 4-5C8, and 4-5G9, were used to magnetically sort PSC-derived islet cells, which led to the formation of islet-like clusters with improved GSIS and reduced growth upon transplantation. Thus, these antibodies selectively isolated islet cell populations from PSC differentiated *in vitro* using a scalable magnetic sorting approach, facilitating the large-scale production of safe and functional islets from stem cells (12).

## Depletion of Contaminant Pluripotent Stem Cells in the Final Cell Product

**FIGURE 1:**
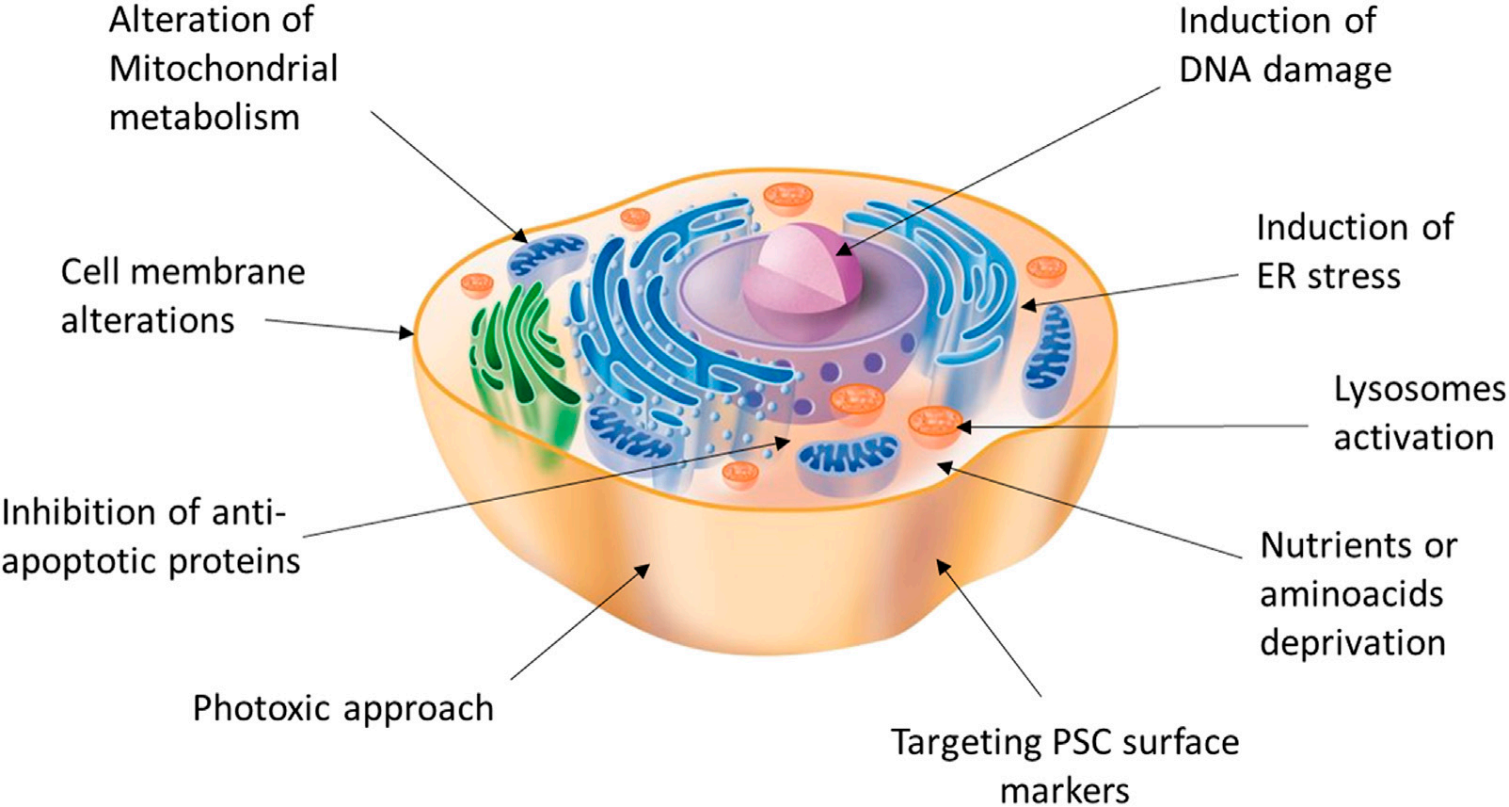
Potential targets and strategies acting on different cell compartments for the induction of selective PSC death.

Despite its efficiency, antibody-mediated cell sorting using surface markers to detect and select pancreatic cells does not guarantee a lack of undifferentiated cells in the sorted group. Moreover, cell sorting is a technique that inherently exerts a strong mechanical stress, which can heavily affect cell viability. However, antibody-mediated strategies could still be combined with other positive selection solutions or even replaced with direct depletion of the contaminant pluripotent cells remaining after the differentiation process. In fact, the two main characteristics of PSC, namely pluripotency and active proliferation capacity, can be exploited for the development of highly selective strategies that facilitate their elimination (48). Therefore, a variety of approaches have been reported, including the use of drugs/phototoxic approaches linked to antibodies targeting PSC surface-specific antigens or small molecules for selective elimination (Figure 1).

### Antibody-Mediated Selection

As previously described, cell sorting using antibodies against specific surface proteins has primarily been used to isolate desirable cell types after differentiation. Alternatively, undifferentiated PSC can be identified by exploiting specific surface marker expression profiles. Antibodies against tumor-related antigen (TRA)-1-60 and TRA-1-81 or stage-specific embryonic antigens (SSEAs), such as SSEA-3, SSEA-4 (49), and SSEA-5 (50) were used to negatively select PSC from a mixed cell population. However, when using magnetic-activated cell sorting (MACS), it was not possible to achieve complete separation, and thus the elimination of undifferentiated ESC, while using highly selective fluorescence-activated cell sorting (FACS), thereby compromising the viability of PSC derivative cells (49). Therefore, the use of an antibody capable of inducing cell death or separation based on a specific surface protein linked to a cytotoxic agent is a valid approach to reduce the potential for teratoma formation in heterogeneously differentiated cultures, as the specificity of antibodies can be exploited without using sorting techniques.

Choo et al. generated 10 monoclonal antibodies against the surface antigens of undifferentiated ESC, showing strong reactivity against undifferentiated, but not differentiated, cells. Among these antibodies, IgM mAb 84, which binds the antigen podocalyxin-like protein-1, was found to be cytotoxic to undifferentiated ESC in a concentration-dependent and complement-independent manner. Single-cell suspensions of undifferentiated ESC pre-treated *in vitro* with mAb 84 before transplantation into mice did not form tumors even 18 weeks after infusion (51). This strategy was later combined with MACS selection with an anti-SSEA-1 antibody for the selective removal of 99.1–100% of undifferentiated ESC (52). One of the main problems associated with this strategy is the large size of mAb 84, which can impede penetration into embryoid bodies (EB) or cell clusters. Consequently, four antibody fragment formats of mAb 84 were engineered and among these only one, scFv 84-HTH, a single chain variable fragment with a dimerizing helix–turn–helix motif, could recapitulate the cytotoxicity of mAb 84 on multiple hESC lines (53).

Another strategy that exploits hyperglycosylated podocalyxin expression is based on the recombinant lectin probe, rBC2LCN. Initially, this molecule was used for fluorescence-based imaging (54) and quantitative detection (55). However, it was later conjugated with a catalytic domain of *Pseudomonas aeruginosa* exotoxin A, which led to the formation of a recombinant lectin-toxin fusion protein, termed rBC2LCN-PE23. rBC2LCN-PE23 binds to human PSC, followed by its internalization, allowing for the intracellular delivery of the cytotoxic protein, which is sufficient to completely eliminate human PSC but not differentiated cells (56). Ben-David et al. also showed that a cytotoxin-conjugated antibody that selectively targets Claudin-6-positive cells efficiently kills undifferentiated cells, thus eliminating the tumorigenic potential of human PSC cultures containing undifferentiated cells, as Claudin-6 is absent in adult tissues but highly expressed in undifferentiated cells (57).

In a recent study, desmoglein 2 (Dsg2), which is highly expressed in undifferentiated PSC versus somatic tissues, was targeted using the monoclonal antibody K6-1 linked to the chemotherapeutic agent doxorubicin (DOX). Dsg2-positive hPSC were selectively targeted by K6-1-DOX, which led to the pH-dependent endosomal release and nuclear localization of DOX, with subsequent cytotoxicity via an apoptotic caspase cascade. The drug is highly efficient in preventing teratoma formation upon iPSC transplantation (58); however, its effect on PSC-derived cells transplanted *in vivo* has not yet been investigated. Conversely, Sougawa et al. proposed a new clinical grade method to eliminate residual undifferentiated iPSC from differentiated cardiomyocyte cell culture using the anti-CD30 antibody-drug conjugate brentuximab vedotin, which selectively kills CD30-positive cells by inducing cell cycle arrest in the G2/M phase followed by apoptosis (59). The researchers demonstrated that undifferentiated iPSC express the surface marker CD30, a TNF receptor superfamily member, at high levels, and brentuximab vedotin treatment induces PSC apoptosis and prevents teratoma formation without affecting the differentiated cardiomyocytes (59). We recently applied this strategy in the field of diabetes, confirming that treatment with brentuximab vedotin efficiently induced cell death in human iPSC while sparing iPSC-derived β cell identity and function. The transplantation of non-treated human iPSC-derived β cells into NOD-SCID mice may result in teratoma formation within 4 weeks, whereas cells treated with brentuximab vedotin prior to transplantation did not result in the formation of teratomas. These findings suggest that targeting the CD30-positive iPSC residual fraction reduces the tumorigenicity of human iPSC-derived β cells, potentially enhancing the safety of iPSC-based β cell replacement therapy (60).

Another strategy for eliminating pluripotent cells is the phototoxic approach. Indeed, in 2003, a new method for selective cell targeting was described, based on the use of light-absorbing microparticles and nanoparticles heated by short laser pulses to create highly localized cell damage (61). This strategy was then applied for the ablation of hPSC from differentiating cell cultures using antibodies directed against the hPSC surface markers Tra-1-60 and Tra-1-81, which were targeted with nanogold particles. Subsequent laser exposure resulted in 98.9 ± 0.9% elimination of hPSC by photothermolysis, while co-treated differentiated cells maintained their normal proliferation and differentiation potential. Moreover, the *in vivo* transplantation of treated mixed hPSCs/differentiated cell cultures revealed that laser ablation can strongly reduce the risk of teratoma formation (62). Alternatively, the PSC-specific fluorescent probe CDy1 was found to induce the selective death of murine and human PSC. CDy1 is a fluorescent rhodamine compound that induces the generation of reactive oxygen species in PSC and determines selective PSC death by simple visible light irradiation, without affecting other differentiated cells. Notably, a single 1 minute exposure of CDy1-stained PSC to visible light confirmed the inhibition of teratoma formation in mice (63).

### Small Molecules

The first report of a small molecule that induced the selective cell death of hESC dates back to 2004, when Bieberich et al. described that, in tumors formed after engraftment of differentiated neuronal cells into the mouse brain, Oct-4 expression co-localized with that of PAR-4, a protein that mediates ceramide-induced apoptosis during neural differentiation of ES cells. They then demonstrated that a ceramide analog, N-oleoyl serinol (S18), can eliminate human Oct4^+^/PAR4^+^ cells and increase the proportion of Nestin-positive neuroprogenitors, and that this enrichment prevents teratoma formation (64). However, this strategy exploits the characteristics of pluripotent cells committed to neuronal differentiation and is therefore not applicable for differentiation into other lineages, including β cells. Instead, a feature common to pluripotent stem cells, which distinguishes them from all somatic cells, is their high susceptibility to DNA damage (65), as PSC commit programmed cell death even under low genotoxic stress to ensure genomic stability (66). This rapid apoptosis process results from the high induction of mitochondria-dependent cell death mechanisms, which can be mediated through several processes, such as cytoplasmic p53, mitochondrial translocation of BAX, or through the inhibition of ESC-specific anti-apoptotic proteins, such as BIRC5 (Survivin) or BCL10 (67). This peculiarity has therefore been widely exploited in research on small molecules capable of inducing the selective death of PSC, since adult stem/progenitor cells express other pro-survival proteins. For instance, it was demonstrated that a single treatment of PSC-derived cells with chemical inhibitors of Survivin, such as the flavonoid quercetin (QC) or YM155, induced the selective and complete cell death of undifferentiated hPSC and prevented teratoma formation, while differentiated cell types derived from PSC survived and maintained their functionality (68,69). Recently, it was reported that another natural flavonoid, luteolin, is even more potent than QC in selectively inducing PSC death in a p53-dependent manner (70). However, the effect of this molecule has not been explored *in vivo*. Similarly, the sequential administration of the mitotic drug Taxol at very low doses followed by the CDK inhibitor purvalanol A has been shown to eliminate Survivin activity; this drug combination was able to induce apoptosis in ESC and teratomas (71), although tissue analysis was performed only 18 h after transplantation and a longer follow-up was not reported. However, the efficacy of purvalanol A for PSC-derived teratoma eradication (together with two CDK1 inhibitors, dinaciclib and Ro-3306) in another study showed that inhibiting CDK1 leads to the activation of the DNA damage response and negative regulation of the anti-apoptotic protein MCL1 in human and mouse ESC, but not in differentiated cells (72). Apoptotic susceptibility to DNA damage in PSC was also tested using the genotoxic anti-tumoral drug etoposide, which effectively purged the population of residual teratoma-forming cells within the progenitor population of cells upon *in vivo* transplantation, without causing genomic instability in the surviving progeny (73). Furthermore, Brequinar, an inhibitor of dihydroorotate dehydrogenase (DHODH), a key enzyme in the *de novo* pyrimidine synthesis pathway, was shown to be effective in inducing cell cycle arrest, cell death, and stemness loss in mouse PSC (74). However, its effect has yet to be evaluated in PSC of human origin.

Using compound screening, the ER stress induction molecule JC011 was found to induce cell death in PSC; undifferentiated cells pre-treated with this compound failed to form teratomas in immunodeficient mice (75). Using a similar approach, the screening of a library of cytotoxic compounds identified methyl 27-deoxy-27-oxookadaate, a substrate for two ATP-binding cassette transporters (ABCB1 and ABCG2) whose expression is repressed in PSC, as a reagent that selectively induces the death of human pluripotent stem cells (76). Similarly, the high-throughput screening of over 50,000 small molecules identified 15 pluripotent cell-specific inhibitors (PluriSIns) (77). Among these, PluriSIn#2 induces PSC selective death by suppressing the expression of topoisomerase, an enzyme essential for maintaining DNA integrity. Notably, topoisomerase IIα (TOP2A) is uniquely expressed in undifferentiated cells and is downregulated during their differentiation. PluriSIn#2 does not directly inhibit TOP2A enzymatic activity, but rather selectively represses its transcription, thereby significantly reducing TOP2A protein levels (78). Doxorubicin, a proven chemotherapeutic agent, is another inhibitor of topoisomerase II that has been shown to increase cardiomyocyte purity by removing potential proliferative stem cells from terminally differentiated cells. Doxorubicin, however, does not discriminate between the two isoforms of topoisomerase II (α, PSC- and cancer-specific, and β, expressed in almost all cell types). Therefore, in this study, it was crucial to determine the optimal doxorubicin dosage that prevented cell proliferation of residual undifferentiated stem cells while being non-cardiotoxic towards more terminally differentiated cells (79). However, although effective, strategies that induce oxidative stress or DNA damage should be carefully evaluated and used with caution, as they may increase the risk of DNA damage in differentiated cell types.

Another possibility that would allow for the selective elimination of pluripotent cells involves taking advantage of the different pathways of PSC compared to differentiated cells. PSC produce most ATP via glycolysis, transitioning to oxidative phosphorylation (OXPHOS) for most ATP production during differentiation (67). Cardiomyocytes, for example, produce the most energy using glucose, fatty acids, and lactate by OXPHOS. It has been demonstrated that these differentiated cells can be purified from PSC using a medium lacking glucose and glutamine, but supplemented with lactate (80). However, many other differentiated cell types cannot uptake and metabolize lactate, making this strategy cell-specific. In particular, this strategy would not be suitable for β cells, as glucose is fundamental for insulin release and β cells lack the lactate transporter MCT13 and have reduced expression of lactate dehydrogenase (81). Similarly, even the use of an inhibitor of glucose transporter 1 (GLUT1) such as STF-31, which is able to selectively kill undifferentiated PSC (82,83), is not applicable to the β cell field, as GLUT1 is the main glucose transporter in human insulin-secreting β cells (84). The response to treatment with high concentrations or deprivation of specific amino acids is also different between undifferentiated PSC and differentiated cells, and these differences may be used for the selective elimination of PSC. For instance, a high concentration of l-alanine was able to selectively eliminate undifferentiated iPSC co-cultured with differentiated cells (85); however, this strategy would not be feasible for PSC-derived insulin-secreting cells, as prolonged l-alanine exposure induces changes in metabolism, Ca^2+^ handling, and desensitization of insulin secretion in pancreatic β cells (86). L-methionine-free media were also tested as a PSC-depleting agent in combination with cell culture at 42°C, demonstrating that this combination of culture conditions is capable of preventing tumor formation upon iPSC subcutaneous transplantation (87). In addition, in this case, the strategy does not seem applicable to β cells, as L-methionine has recently been shown to prevent β cell damage and modulate the β cell identity marker MafA (88).

The most selective compound for achieving PSC-specific selective killing among the PluriSIns identified is PluriSIn#1, an inhibitor of stearoyl-coA desaturase (SCD1), which catalyzes the conversion of saturated fatty acids to monounsaturated fatty acids. Even if the expression level of SCD1 in PSC is comparable with that of other cell types, the biosynthesis of oleate by SCD1 is a vital process in PSC, which is highly sensitive to SCD1 inhibition. As a result, PluriSIn#1 activates a cascade of events that culminate in the death of these cells via apoptosis after the induction of ER stress, mitochondrial ROS, and mitochondrial DNA damage. The treatment of a mixed population of pluripotent and differentiated cells for 48 h with PluriSIn#1 was reported to prevent teratoma formation in mice (77). However, the researchers did not show the analysis of the grafts of the animals that did not develop teratomas and did not confirm that the differentiated cells were the only ones to have survived.

Another molecule capable of acting on mitochondrial metabolism is MitoBloCK-6, an inhibitor of the mitochondrial redox protein Erv1/ALR, which induces apoptotic cell death via the selective release of cytochrome C in PSC, but which has no effect on differentiated cells (89). However, it remains unclear how PSC are specifically sensitive to MitoBloCK-6. Similarly, the mechanism of action of metformin, which has been shown to be effective in preventing the occurrence or in decreasing the size of teratomas after transplantation of iPSC in an apoptosis-independent manner, has not yet been elucidated. The hypothesis is that metformin suppresses the expression of Oct4 and Survivin, two pivotal genes of malignant stem cells responsible for teratocarcinoma growth, circumventing the suppression of AMPK (AMP-activated protein kinase (AMPK), which allows iPSC to avoid anabolic inhibition, similar to cancer cells (90).

Molecules capable of targeting various other elements of the cell, such as lysosomes, proteins, and pumps present on the cell membrane, have also been described. Recently, Chakraborty et al. explored the use of WX8 and apilimod as inhibitors of PIKfyve phosphatidylinositol kinase, which is essential for lysosome homeostasis, to selectively kill PSC under conditions where differentiated cells remain viable (91). PIKfyve inhibitors prevent lysosome fission, induce autophagosome accumulation, and reduce cell proliferation in both pluripotent and differentiated cells, but induce death specifically in pluripotent cells by non-canonical apoptosis (91). Recently, it has been shown that bee venom (BV) can specifically induce cell death in iPSC but not in iPSC-derived differentiated cells; however, the cause of this selectivity has yet to be clarified. BV was found to rapidly disrupt cell membrane integrity and focal adhesions, followed by the induction of apoptosis and necroptosis in iPSC, with BV exposure remarkably enhancing intracellular calcium levels, calpain activation, and reactive oxygen species generation (92).

In another study, the cytotoxic effects of the US Food and Drug Administration (FDA)-approved cardiac glycosides (CG), such as digoxin and lanatoside C, on ESC were investigated (93). CG is a specific inhibitor of the transmembrane sodium pump Na^+^/K^+^-ATPase, which leads to an increase in the intracellular concentrations of calcium ions. ESC expressed Na^+^/K^+^-ATPase more abundantly than adult stem cells. Thus, the viability of the ESC-derived cells was not affected by digoxin and lanatoside C treatments. Furthermore, *in vivo* experiments have demonstrated that digoxin and lanatoside C prevent teratoma formation (93).

In general, there are no single small molecules suitable for all types of differentiation, as these compounds often exploit the biological properties of pluripotent cells potentially shared by differentiated cells (i.e., JC011 and 27-deoxy are toxic for neurons, and MitoBloCK-6 is toxic for cardiac development). Notably, all the depletion approaches presented thus far have proven to be effective in selectively killing PSC without damaging the differentiated cells and preventing or limiting teratoma formation. However, despite their proven efficacy, almost none of these strategies have been tested in PSC-derived β cells for diabetes cell therapy. In addition, there is also a need to develop an alternative safe approach to selectively eliminate PSC *in vivo* after accidental transplantation into patients. To this end, genome editing strategies may be a solution to this critical problem.

## A Safety Switch for a Safer Cell Therapy

**FIGURE 2:**
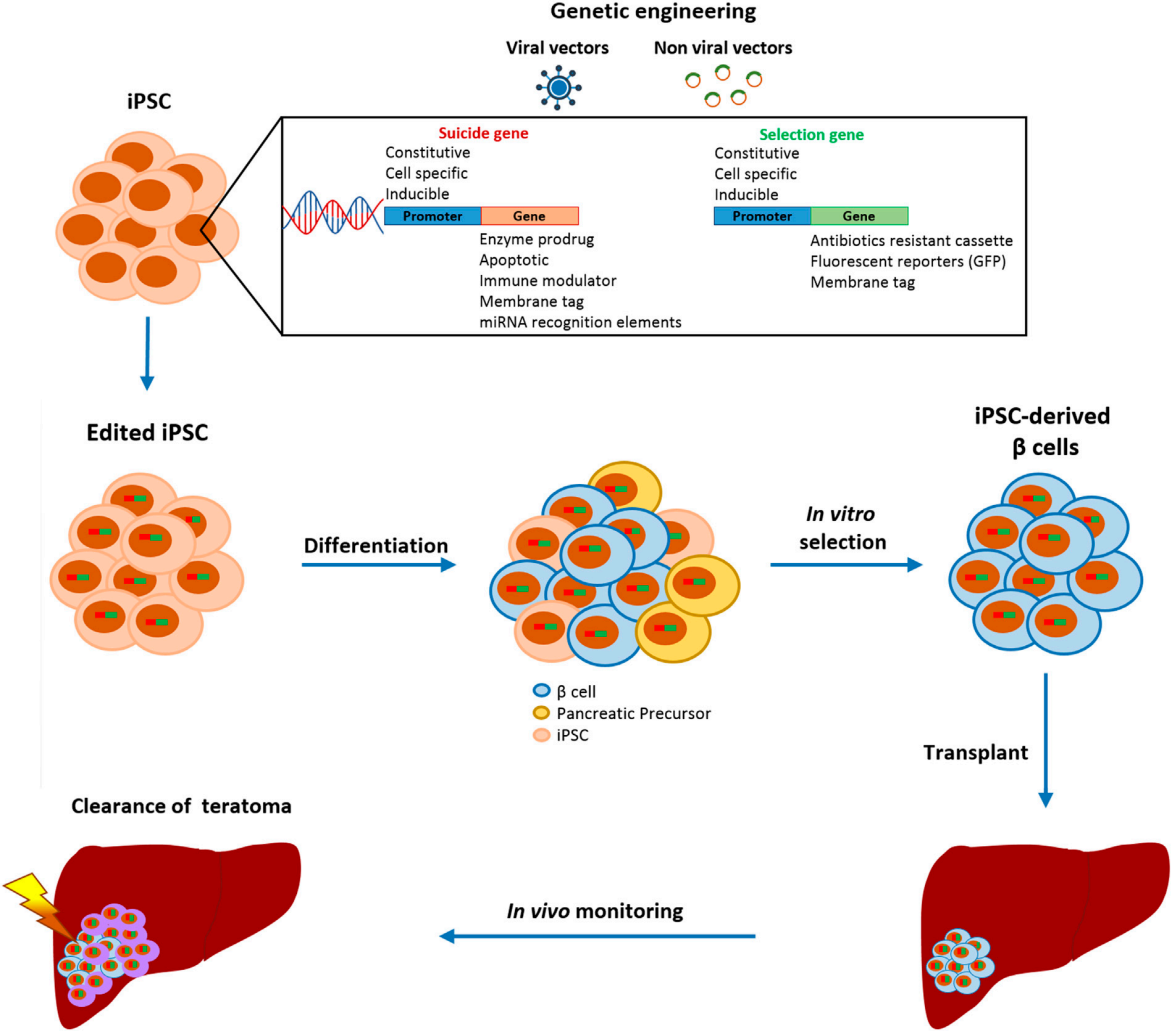
Schematic representation of gene editing strategies to increase the safety of iPSC-derived β cell transplantation. Gene-edited iPSC are differentiated into β cells and only insulin-positive cells are purified.

One strategy to fully control the cellular product, even after transplantation, is to equip cells with a suicide gene that can eliminate cells that have gone astray, since mutations can occur anytime and differentiated cells can undergo malignant transformation *in vivo* (Figure 2) (94). Ideally, the insertion of a suicide gene, which can be stably expressed in both quiescent and replicating cells, should not impair the pluripotency, differentiation, or genomic stability of PSC (95). The choice of the gene editing approach is based on the type of target cells that will be edited. Since gene editing of hESC or iPSC has a lower efficiency rate compared to other cell lines due to lower resilience to DNA damage (96), protocols designed *ad hoc* for human PSC must be adopted. The crucial components to be evaluated for the efficient gene editing of PSC are the choice of the delivery vectors and of the suicide genes with their relative selection marker, as the selection of edited cells is fundamental to obtain a pure edited population. Selection methods include the addition of antibiotic-resistant cassettes or genome-edited cell sorting based on the induced expression of fluorescent reporters or surface antigens (90–92). These selection strategies can also be combined to obtain a purer population or to select a cell population edited with more than one construct.

### Viral and Non-viral Vectors

The vector is essential for the delivery of gene constructs to PSC. Currently, both viral and non-viral gene delivery systems are used to this end.

Among the most common viral delivery systems, retrovirus (RV), lentivirus (LV), Epstein-Barr virus (EBV), herpes simplex virus (HSV), and baculovirus (BV) have a higher transduction efficiency for PSC than adenovirus (AV) and adeno-associated virus (AVV) (97,98). In particular, RV (99) and LV (100) have the highest transduction efficiency; however, they permanently modify the host genome with the risk of causing insertional mutations when randomly incorporated (101). Conversely, EBV (102), HSV (103) and BV (104) are non-integrating viruses that mediate transient gene expression in dividing and non-dividing cells (97). Additionally, AV does not integrate into the host genome and allows long-term transgene expression, as AV persists as an episome in the nucleus. However, due to the active cell division or proliferation of PSC, the percentage of transduced cells decreases over time (105,106). Viral gene delivery systems are primarily based on DNA, RNA, and oncolytic vectors. The vectors based on DNA deliver a plasmid containing the gene construct (107), while the RNA-based vectors provide RNA-dependent RNA polymerase complexes coupled with negative-strand RNA templates (108). The oncolytic vectors, an emerging weapon in the cancer field, are able to specifically target and lyse tumor cells (109).

Non-viral gene systems allow for construct delivery via physical or chemical methods, including electroporation or liposomes, which show less toxicity and immunogenicity than viral vectors; however, their transfection efficiency is orders of magnitude lower than that of viral vectors (110). Among the non-viral gene delivery systems described thus far, the scaffold/matrix attachment regions (SMARs) are non-integrating vectors suitable for PSC engineering and can autonomously replicate without causing molecular or genetic damage. Moreover, SMARs provide sustained transgene expression during the reprogramming and differentiation of PSC and their progenies (111).

Regardless of the strategy used, if the chosen vector integrates the genetic material, the insertion site is of fundamental importance, as random gene insertion may lead to perturbation of endogenous gene activity and the inactivation of a random gene, leading to the death of the targeted cell or cancer promotion (112). Thus, the installation of the suicide switch into a genomic safe harbor is fundamental for the establishment of a safe and efficient system. Among the known safe harbors in the human genome, namely AAVS1, CCR5, and the human homolog of murine ROSA26 locus, AAVS1 is the most studied for PSC gene editing, as no gross abnormalities or differentiation deficits were observed in PSC harboring transgenes targeted in AAVS1 (112,113). Moreover, transgene expression at this locus is stable and consistent across different cell types (112,114).

To date, site-specific genome editing can be achieved by applying zinc-finger nucleases (ZFN), transcription activator-like effector nucleases (TALEN), or CRISPR/CAS9 systems (115). ZFN and TALEN are based on similar principles: they contain a FoxI endonuclease and exploit protein-DNA binding. Although both TALEN and ZFN have been applied for genome editing in human PSC (116,117), the ZFN system is the worst in terms of target specificity and off-target frequency (118). The more novel CRISPR system contains a Cas9 nuclease and has a binding principle based on RNA-DNA. Compared to ZFN and TALEN, the CRISPR/Cas9 system has the highest target specificity and lower off-target frequency (118,119). Therefore, CRISPR/Cas9 is becoming the most used system for the genetic manipulation of hPSC (120).

### Suicide Genes

The choice of the gene and promoter to be used for its expression is of crucial importance for efficient gene editing. The most efficient and widely used suicide gene is herpes simplex virus thymidine kinase (HSV-TK), which induces apoptosis in edited cells upon treatment with ganciclovir (GCV) by inhibiting DNA synthesis (85). Schuldiner et al. were the first to demonstrate that using GCV enables the *in vivo* elimination of a teratoma originating from the injection of edited ESC into SCID mice using cells edited with a constitutive promoter, PGK, carrying the expression of the HSV-TK gene (121). However, this strategy is not applicable for selectively removing pluripotent undifferentiated cells from a heterogeneous cell preparation, as a constitutive promoter leads to the constitutive expression of the target gene in all undifferentiated and differentiated cells. Consequently, another possibility involves the selection of a promoter that targets a gene specifically expressed by PSC, enabling the survival of differentiated progenitors. For example, adding a suicide gene under *TERT, OCT4*, *TERF1*, or *NANOG* promoters, which are highly expressed in a pluripotent state, can selectively remove undifferentiated cells (122–125).

The most commonly used suicide genes perform their function via enzymatic drug conversion activity, apoptotic potential, or the ability to direct the immune response against a cell by the addition of a tag (126). In particular, enzyme prodrugs can enzymatically convert an innocuous prodrug into a toxic compound that can kill the target cell (127). The toxic molecule generated can act only towards the edited cell or can have a broader action killing also the surrounding cells, called the “bystander effect,” usually used to treat cancer (128). The most common prodrug enzymes used are HSV-TK, cytosine deaminase (CD) from *Escherichia coli* or yeast, and *E. coli*-associated nitroreductase (NTR), which make cells sensitive to the prodrugs GCV, 5-fluorocytosine (5-FC), and CB1954, respectively (127).

HSV-TK, CD, and NTR have been used as safety switches in the field of PSC (122,129,130), among which HSV-TK is one of the most studied and applied prodrug-activated enzymes (131). Interestingly, Rong et al. introduced the HSV-TK gene into the 3′-untranslated region of the endogenous NANOG gene in ESC and found that the safety switch allowed for the clearance of residual undifferentiated cells from differentiated neural populations *in vitro* and *in vivo* in an SCID mouse model (132). Another possibility involves introducing HSV-TK into human and murine ESC under the control of a cell division gene, such as *CDK1*, which is fundamental for the G2 to M phase transition. Specifically, Liang et al. introduced HSV-TK into the *Cdk1* 3′-untranslated region in homozygosity, which allowed for the maintenance of CDK1–TK expression without incurring a loss of gene function due to mutational events. Upon the transplantation of edited ESC-derived neural epithelial progenitors into mice, only the proliferative cells died after GCV administration, leaving the non-dividing differentiated cells intact (133). This approach may be interesting for application in PSC-derived β cells, as the final differentiated cell no longer has proliferation capacity compared to the PSC and progenitor cells. The miRNA regulatory system can also be used in suicide gene therapy strategies. For instance, the specific expression of the let7 miRNA family in differentiated cells, but not in pluripotent cells, has been exploited to construct an HSV-TK gene under the constitutive promoter human translation elongation factor 1A (EF1α) tagged to four tandem miRNA recognition elements (MRE) complementary to mature miRNAs of the let7 family. In this case, HSV-TK was specifically expressed in PSC that were selectively killed by GCV, whereas differentiated cells were fully protected (134).

Despite its effectiveness in killing target cells, some disadvantages of the HSV-TK system include immunogenicity, *in vivo* drug resistance, and the presence of inactivating mutations (94,134,135). Moreover, a recent study documented the acquisition of GCV resistance by iPSC expressing HSV-TK (87), underlining the need for the use of gene editing techniques that allow for insertion in genomically safe harbors that cannot be silenced.

Notably, a recent study on ESC-derived β cells applied a double fail-safe approach, capable of both killing residual PSC and selecting insulin-positive cells (130). Specifically, they used the HSV-TK cassette placed under the human telomerase reverse transcriptase (hTERT) promoter, which is highly expressed only by stem cells and tumor cells, to induce PSC-selective death when exposed to GCV. At the same time, nitroreductase (NTR) was used to select insulin-positive cells, as this construct is flanked by *loxP* sites and eliminated by Cre expression under the control of the human insulin promoter. Therefore, insulin-expressing cells are rendered insensitive to the prodrug CB1954. Using this method, only insulin-positive and non-proliferating cells survive selection, and cells that may de-differentiate after transplantation may still be selectively killed *in vivo* by GCV without affecting the rest of the graft (130).

Suicide genes, with apoptotic potential, are directly involved in triggering the apoptotic pathway. The most known are Fas ligand, Fas, FADD, caspase-3, caspase-8, caspase-9, p53, p33ING1, p73α, Bax, Apaf-1, IkappaBdN, Bcl-2, Bcl-x, and NBK (126), some of which have also been used to eliminate pluripotent cells. For instance, the inducible caspase-9 (iCASP9) suicide gene, under the control of the endogenous *OCT4* promoter, was applied to specifically kill undifferentiated PSC *in vitro* and *in vivo* (136). Similarly, the *SOX2* promoter has been exploited as a safeguard system for PSC-based therapies (137). However, *SOX2* is a less specific marker since it is also expressed in differentiated lineages, including ectoderm and endoderm (138,139). Thus, this strategy could not be applied to the β cell replacement field. Another study in iPSC used iCASP9 under the control of a constitutive promoter EF1α, which is able to eliminate pluripotent cells within 24 h of exposure to a chemical inducer of dimerization, AP20817 (140). Similarly, iCASP9, under the control of the synthetic promoter CAG, allowed for the killing and complete elimination of iPSC *in vitro* by inducible activation using AP1903, a lipid-dependent tacrolimus analog. In this case, a synthetic promoter was chosen to obtain higher expression levels (141).

Recently, a new drug-inducible safeguard combination has been adopted to eliminate *in vitro* and *in vivo* undifferentiated PSC. The construct NANOG-iCASP9, activated by the AP20187 molecule, has been used to induce PSC apoptosis and reduce the risk of teratoma formation prior to transplantation, while the construct ACTB-OiCASP9, activated by AP21967, killed all PSC-derived cell types to protect against *in vivo* adverse events. A third construct, ACTB-HSV-TK, activated by GCV, was used to kill all PSC-derived dividing cells *in vivo* (142). The iCASP9 suicide gene system is effective, safe, and less immunogenic owing to its human origin (143).

Another strategy to reduce the tumorigenic potential of ESC and iPSC involves exploiting the antitumor function of p53, which increases the gene copy number while retaining full pluripotency. Edited cells showed an improved response to anticancer drugs, which could aid in their elimination when tumors arise (144). Moreover, enzymatic activity already present in the cell, such as alkaline phosphatase (ALP), particularly overexpressed by iPSC, can be exploited to selectively kill pluripotent cells. The peptide l-phosphopentapeptide, when dephosphorylated by ALP, forms intranuclear peptide assemblies that lead to cell death, but is innocuous to normal cells, which do not overexpress ALP (145).

Taking advantage of the recipient immune system represents another strategy to selectively kill a target cell. For instance, porcine xenoantigen α1,3-galactosyltransferase (GalT) was inserted under the control of the hTERT promoter in hESC. As in human serum, antibodies against the α-gal epitope and GalT expression are present only in edited PSC, and the immune system directly kills hESC upon transplantation, providing protection from *in vivo* cell dedifferentiation or *de novo* tumor formation that involves hTERT reactivation (146).

Marking cells with a distinctive tag expressed in the plasma membrane represents another method available in the field of suicide gene technology. The tag should preferably not be an immunogenic human sequence. This approach, which is mostly used in T cell transplantation, allows for the *in vivo* control of adverse events associated with the use of stem cell-derived differentiated cells. For instance, the overexpression of the CD20 tag has been assessed in combination with an anti-tag monoclonal antibody, which can be administered *in vivo*, for an antibody-dependent cytotoxic response (147). However, this strategy has yet to be applied to iPSC (148). One possible disadvantage of this method is that it incurs a toxic off-target effect if the antibody binds other cells that express the same receptor. Finally, a new frontier is the application of an engineered oncolytic virus to selectively replicate in and kill tumor cells. Mistui et al. developed a conditionally replicating adenovirus (CRA), and in particular, a variety of CRAs, such as Surv.m-CRA and Tert.m-CRA, that replicate only in undifferentiated cells as they are controlled by the Survivin and *TERT* promoters, which are more expressed in PSC (149).

In conclusion, gene editing represents a promising approach for the control PSC-derived cellular products, especially with regards to the elimination of cells with tumorigenic potential *in vitro* and intervening in time in case of tumor occurrence *in vivo*. Currently, there are many preclinical and clinical studies that confirm the validity of this approach. In general, for PSC gene editing, a vector capable of providing a stable and efficient insertion must be chosen because the stability of the insertion should be maintained in the pluripotent state and in the progeny, during cellular differentiation, and in the final differentiated stage. Moreover, accuracy is required to select the best promoter-gene construct expressed only in the target cell population, which does not undergo silencing, reduction of expression, or changes due to mutations. In addition, issues related to immunogenicity and toxicity of the inserted genes must also be considered.

## Discussion

A new source of insulin-producing cells would represent a significant step forward in cell therapy for the treatment of diabetes. Stem cells are strong candidates due to their infinite replication and differentiation capabilities, as well as their ability to be gene-edited. Among stem cells, iPSC are of particular interest because they can be derived from any individual, and there are numerous *in vitro* differentiation protocols capable of transforming them into β cells in an efficient and reproducible manner. Within the context of the use of iPSC-derived β cells in clinical applications, safety issues are an essential consideration. In this regard, we have identified and described four main steps to ensure the transplantation of safe cellular product in patients. First, iPSC must be reprogrammed with a non-integrating vector that is easily cleared from the cell, such as the latest generation of Sendai viruses. Second, the differentiated β cells must be purified as much as possible using the surface markers identified or in combination, such as GP2 at the precursor stage and CD49a at the β cell stage. However, if this selection is not 100% effective, treatments with molecules or antibodies that eliminate the residual stem component could be employed, for example adding PluriSIns or anti-CD30 monoclonal antibody to the iPSC derivatives. Finally, it is desirable to incorporate a suicide gene into iPSC, enabling the conversion of a non-toxic prodrug into an active cytotoxic compound that kills the cell itself. In this case, if tumor cells develop after transplantation, the graft can be eliminated by prodrug assumption. However, it must be taken into consideration that with the use of gene editing strategies, cell therapies will require further regulatory review steps to ensure patient safety.
